# Circulating pancreatic cancer exosomal RNAs for detection of pancreatic cancer

**DOI:** 10.1002/1878-0261.12398

**Published:** 2018-11-15

**Authors:** Tatsuya Kitagawa, Keisuke Taniuchi, Makiko Tsuboi, Masahiko Sakaguchi, Takuhiro Kohsaki, Takehiro Okabayashi, Toshiji Saibara

**Affiliations:** ^1^ Department of Gastroenterology and Hepatology Kochi Medical School Kochi University Nankoku Japan; ^2^ Department of Endoscopic Diagnostics and Therapeutics Kochi Medical School Kochi University Nankoku Japan; ^3^ Department of Integrated Center for Advanced Medical Technologies Kochi Medical School Kochi University Nankoku Japan; ^4^ Cancer Prevention and Control Division Kanagawa Cancer Center Research Institute Yokohama Japan; ^5^ Department of Surgery Kochi Health Sciences Center Japan

**Keywords:** diagnostic marker, exosome, pancreatic cancer, small nucleolar RNA

## Abstract

Diagnostic biomarkers for the early diagnosis of pancreatic cancer are needed to improve prognosis for this disease. The aim of this study was to investigate differences in the expression of four messenger RNAs (mRNAs: *CCDC88A*,*ARF6*,* Vav3*, and *WASF2*) and five small nucleolar RNAs (snoRNAs: *SNORA14B*,*SNORA18*,*SNORA25*,*SNORA74A*, and *SNORD22*) in serum of patients with pancreatic cancer and control participants for use in the diagnosis of pancreatic cancer. Results were compared with the expression of sialylated Lewis (a) blood group antigen CA19‐9, the standard clinical tumor biomarker. Reverse transcription quantitative real‐time PCR showed that all of the mRNAs and snoRNAs, except *CCDC88A*, were encapsulated in exosomes and secreted from cultured pancreatic cancer cells, and present in cell culture medium. In a discovery‐stage clinical study involving 27 pancreatic cancer patients and 13 controls, the area under the receiver operating characteristic curve (AUC) of two mRNAs (*WASF2* and *ARF6*) and two snoRNAs (*SNORA74A* and *SNORA25*) was > 0.9 for distinguishing pancreatic cancer patients from controls; the AUC of CA19‐9 was 0.897. Comparing serum levels of *WASF2*,*ARF6*,*SNORA74A*,*SNORA25*, and CA19‐9 revealed that levels of *WASF2* were the most highly correlated with the risk of pancreatic cancer. The AUCs of *WASF2*,*ARF6*,*SNORA74A*, and *SNORA25* in serum from patients in the early stages of pancreatic cancer (stages 0, I, and IIA) were > 0.9, compared with an AUC of 0.93 for the level of CA19‐9. The results of this study suggest that *WASF2*,*ARF6*,*SNORA74A*, and *SNORA25* may be useful tools for the early detection of pancreatic cancer. Monitoring serum levels of *WASF2* mRNA may be particularly useful, as it was the most highly correlated with pancreatic cancer risk.

**Abbreviations**
AUC: area under the receiver operating characteristic curve
CI: confidence interval
lncRNA: long noncoding RNA
mRNA: messenger RNA
OR: odds ratio
PDAC: pancreatic ductal adenocarcinoma
ROC: receiver operating characteristic
snoRNA: small nucleolar RNA

## 1 Introduction

Pancreatic ductal adenocarcinoma (PDAC) is the fourth leading cause of cancer‐related mortality in the Western world (Siegel *et al*., 2013). However, no biomarkers that identify patients with PDAC in the early stages are available (Locker *et al*., 2006), and the absence of reliable serum biomarkers for PDAC reduces the potential effectiveness of screening strategies in at‐risk populations, such as patients with diabetes mellitus and chronic pancreatitis. Therefore, better diagnostic markers of PDAC could improve the early diagnosis of this disease and enable more patients to undergo curative surgical resection.

Exosomes are extracellular vesicles (40–150 nm in size) secreted from all cell types (Thery *et al*., 2002). Endosomes formed during the inward budding of late endosomes can develop into intracellular vesicular endosomes containing messenger RNA (mRNA), microRNA, DNA, long noncoding RNA (lncRNA), RNA‐binding proteins, and lipids (Mathivanan and Simpson, 2009). Recent studies have demonstrated that RNAs, including mRNAs, microRNAs, and lncRNAs, are secreted from tumor cancer cells into body fluids such as blood, urine, milk, and saliva via exosomes (Laurent *et al*., 2015). Exosome concentrations are increased in the systemic circulation of PDAC patients (Nuzhat *et al*., 2017). Recent publications have revealed that exosomes can play diagnostic and prognostic roles in a variety of cancers (Liu *et al*., 2016; Rodríguez *et al*., 2015).

Small nucleolar RNAs (snoRNAs) are noncoding regulatory RNAs of approximately 60–300 nucleotides localized primarily in the nucleolus, where they function in pre‐ribosomal RNA modification and processing (Lafontaine, 2015). Two types of snoRNAs have been described: C/D‐box and H/ACA‐box (Falaleeva and Stamm, 2013). As snoRNAs exhibit tissue‐specific expression (Castle *et al*., 2010), they could serve as novel cancer biomarkers (Su, 2016). The *SNORD50A*/*SNORD50B* locus is deleted in 10–40% of PDAC patients, and the loss of this locus is associated with shorter survival time (Siprashvili *et al*., 2016). Levels of *SNORD33*,* SNORD66*, and *SNORD76* are significantly elevated in the serum of lung cancer patients compared with cancer‐free controls, suggesting they could serve as biomarkers for the early detection of this disease (Liao *et al*., 2010). *SNORA42* is frequently overexpressed in lung cancer and colorectal cancer, and knockdown of *SNORA42* slows the growth of cancer cells, indicating that *SNORA42* is a putative oncogene (Mei *et al*., 2012).

The aim of the present study was to assess the utility of several serum mRNAs (*CCDC88A*,* ARF6*,* Vav3*, and *WASF2*) and snoRNAs (*SNORA14B*,* SNORA18*,* SNORA25*,* SNORA74A*, and *SNORD22*) as diagnostic markers for differentiating PDAC patients from control patients without pancreatic disease. We also describe the differential expression of these mRNAs and snoRNAs in serum samples from patients with early‐stage (0, I, and IIA) and late‐stage (IIB, III, and IV) PDAC.

## 2 Materials and methods

### 2.1 Cell culture

The S2‐013 human PDAC cell line, a subline of SUIT‐2, and HPNE immortalized normal pancreatic epithelial cells were cultured as previously described (Taniuchi *et al*., 2011).

### 2.2 Antibodies

Anti‐CD63 antibody (sc‐15363) was purchased from Santa Cruz Biotechnology (Santa Cruz, CA, USA). Anti‐α‐tubulin antibody (017‐25031) was purchased from Wako Pure Chemical Industries, Ltd. (Osaka, Japan).

### 2.3 RNA fluorescence *in situ* hybridization

S2‐013 cells were fixed in 8% formaldehyde, dehydrated in ethanol (50–70–100%), and held at 4 °C overnight. After cells were rehydrated, permeabilized, and hybridized, fluorescence *in situ* hybridization against four mRNAs (*CCDC88A*,* ARF6*,* Vav3*, and *WASF2*) and five snoRNAs (*SNORA14B*,* SNORA18*,* SNORA25*,* SNORA74A*, and *SNORD22*) was performed using the QuantiGene ViewRNA plate‐based assay kit (Panomics, Santa Clara, CA, USA) according to the manufacturer's recommendations, with some modifications (Taniuchi *et al*., 2014a,b; Taylor *et al*., 2010). Sections were mounted in Aqua‐Poly/Mount (Polysciences, Warrington, PA, USA), and confocal fluorescence images were captured with a VK‐X1000 microscope (Keyence, Osaka, Japan).

### 2.4 Preparation of conditioned medium

S2‐013 and HPNE cells grown to 70% confluence were incubated for 48 h in the presence of conditioned medium supplemented with 10% exosome‐free FBS (Exo‐FBSHI; System Biosciences, Palo Alto, CA, USA). Culture conditioned medium was collected and centrifuged using a Beckman Coulter Allegra X‐15R centrifuge (Brea, CA, USA) at 300 ***g*** and 4 °C for 10 min, and the supernatant was collected as the conditioned medium.

### 2.5 Isolation of exosomal RNAs from cell lysates and conditioned medium

S2‐013 and HPNE cells were lysed in lysis buffer [50 mm Tris (pH 7.4), 150 mm NaCl, 1 mm MgCl_2_, 0.5% NP‐40, a protease inhibitor cocktail tablet (Roche Applied Science, Penzberg, Germany), and phosphatase inhibitor cocktail (Nacalai, Kyoto, Japan)]. The exosomes in the cell lysates were precipitated using an ExoCap Exosome Composite kit (JSR Life Sciences, Tokyo, Japan) according to the manufacturer's recommendations. Exosomes in conditioned medium harvested from S2‐013 and HPNE cells were precipitated using ExoQuick‐TC exosome precipitation solution (System Biosciences) according to the manufacturer's recommendations. RNA was extracted from exosomes isolated from either cell lysates or cell culture medium using a Plasma/Serum RNA Purification Mini kit (Norgen BIOTEK, Thorold, ON, Canada) according to the manufacturer's instructions. The exosomal RNA concentration was determined using a NanoDrop spectrophotometer (Thermo Scientific, Fremont, CA, USA).

### 2.6 Serum samples for reverse transcription quantitative real‐time PCR

Serum samples from patients undergoing resection for PDAC were prospectively obtained at the Department of Surgery of Kochi Health Sciences Center between April 2015 and March 2016. Serum samples from PDAC patients were selected according to the following criteria: (a) Patients were newly diagnosed and previously untreated, (b) tumors were pathologically diagnosed as PDAC, and (c) PDAC patients were not suffering from any kind of malignancies, including PDAC. Tumors were classified (stages I–IV) according to the system of the International Union against Cancer (Table 1; Sobin *et al*., 2009). Clinicopathologic parameters were classified according to pancreatic carcinoma criteria of the Japan Pancreas Society (2003). Control serum samples from individuals diagnosed with benign gastrointestinal diseases who were being evaluated for nonpancreatic diseases were prospectively obtained at the Department of Gastroenterology and Hepatology of Kochi Medical School Hospital. This study was approved by the ethical review boards of Kochi Medical School (approval number: ERB‐101894) and Kochi Health Sciences Center (approval number: 151002) regarding patient recruitment. The study was carried out in accordance with the approved guidelines. Informed consent was obtained from each patient. The experiments were undertaken with the understanding and written informed consent of each subject. The study methodologies conformed to the standards set by the Declaration of Helsinki. Serum was obtained at the time of diagnosis and stored at −80 °C.

**Table 1:** Summary of characteristics of PDAC patients and control patients. IQR, interquartile range

Characteristics	PDAC, % (*n*)	Control, % (*n*)
Age		
≤ 39	0 (0)	0 (0)
40–49	7.4 (2)	7.7 (1)
50–59	11.1 (3)	15.4 (2)
60–69	33.3 (9)	69.2 (9)
70–79	37.1 (10)	0 (0)
≥ 80	11.1 (3)	7.7 (1)
Gender		
Male	63.0 (17)	30.8 (4)
Female	37.0 (10)	69.2 (9)
Diagnosed with diabetes		
Yes	48.1 (13)	23.1 (3)
No	51.9 (14)	76.9 (10)
Diagnosed with hypertension		
Yes	40.7 (11)	38.5 (5)
No	59.3 (16)	61.5 (8)
Serum uric acid		
Upregulated	7.4 (2)	30.8 (4)
Normal range	92.6 (25)	69.2 (9)
Serum triglyceride		
Upregulated	29.6 (8)	30.8 (4)
Normal range	70.4 (19)	69.2 (9)
Stage^a^		
0	0 (0)	
IA	3.7 (1)	
IB	0 (0)	
IIA	25.9 (7)	
IIB	59.3 (16)	
III	11.1 (3)	
IV	0 (0)	
Serum CA19‐9, median (IQR)	195.8 (0.3–24 440)	6.318 (0–132.6)

^a^Classified according to the classification of International Union against Cancer.

### 2.7 Isolation of exosomal RNAs from serum samples

Exosomal RNA was extracted from all serum samples using a Plasma/Serum RNA Purification Mini kit (Norgen BIOTEK) according to the manufacturer's recommendations. The RNA concentration was determined using a NanoDrop spectrophotometer (Thermo Scientific).

### 2.8 One‐step SYBR Green I real‐time RT‐PCR assay

Total RNA from S2‐013 was extracted using an RNeasy kit (Qiagen, Valencia, CA, USA) according to the manufacturer's instructions, and the RNA concentration was determined using a NanoDrop spectrophotometer (Thermo Scientific). One‐step SYBR Green I real‐time RT‐PCR assay (SYBR Green I assay) was performed using a One‐Step TB Green PrimeScript RT‐PCR Kit II (Takara BIO, Shiga, Japan) in a 10‐μL reaction consisting of 1 μL exosomal RNA or total RNA from S2‐013, 0.4 μL each of 10 μm forward and reverse primers, 0.4 μL of PrimeScript One Step Enzyme Mix 2, 0.2 μL of 50× ROX Reference Dye, 5 μL of 2× One Step SYBR RT‐PCR Buffer 4, and 2.6 μL of RNase‐free H_2_O. The primers used for the SYBR Green I assay are summarized in Table 2. For the negative controls, exosomal RNA was substituted with actin beta (*ACTB*) and hypoxanthine phosphoribosyltransferase 1 (*HPRT1*). Forty cycles of amplification were performed using a thermal cycling profile of 94 °C for 15 s, 58 °C for 15 s, and 72 °C for 1 min. Subsequently, a melting curve was recorded by holding at 95 °C for 15 s, cooling to 60 °C for 1 min, and then heating at 0.1 °C·s^−1^ to 95 °C. The amplification and melting curve data were collected and analyzed using stepone software v2.2 (Applied Biosystems, Foster City, CA, USA).

**Table 2:** Primer sequences for the SYBR Green I assay

Gene	Forward primer sequence (5′–3′)	Reverse primer sequence (5′–3′)
*CCDC88A*	CGCAGGAGGACATAGAACCAC	ATGAAGAGGCATGGGGTAGAAA
*ARF6*	TCGCTGGTGATATCCAGATCCTA	ATAGGAACCAGATGCTGCTTTACAA
*Vav3*	CCCATTCAAGGCAGTCAAGTTA	TCTTGTCAGAACACAACTTCTGCTA
*WASF2*	GTGCCAGCTTGGACAGATTGA	GGACACGGTGGGAATGCTTA
*SNORA14B*	CCCTCTTGGTAGCTTCGTTC	CGCAGGTATGAAATAAGACTGAG
*SNORA22*	TTGCACAGTGAACACCCAAGT	AGAGGAGAAGAGCAGGCAATG
*SNORA25*	GGGTCATTTCAAAGAGGGCTTAT	TGGCTTCCTATAGAGAACTTCCATC
*SNORA74A*	TGTCAGCTATCCAGGCTCA	CCCAAAGGTACTCAGCTACAAC
*SNORD22*	ATGTCTTACTCTCTGTCCTAGTCC	ATCCCTCAGACAGTTCCTTCT

### 2.9 Statistical analysis

For *in vitro* experiments, statflex software (ver. 6; YUMIT, Osaka, Japan) and sas software (ver. 9.1.3; SAS Institute, Cary, NC, USA) were used for statistical analyses. Student's *t*‐test was used for comparing continuous variables. *P*‐values < 0.05 were considered significant, and all tests were two‐tailed.

In the discovery‐stage clinical study, statistical analyses were performed using r software (ver. 3.3.3; The R Foundation, Vienna, Austria). The pROC package (The R Foundation) was used for receiver operating characteristic (ROC) curve analysis. The Wilcoxon rank‐sum test was used to compare differences in serum levels of the snoRNAs, mRNAs, and CA19‐9 between the PDAC and control groups. The predictive performance of the snoRNAs and mRNAs was evaluated using ROC analysis, area under the curve (AUC), and corresponding 95% confidence intervals (CIs; DeLong *et al*., 1988) and compared with CA19‐9. Multivariate logistic regression was used to establish the diagnostic mathematical model, and Pearson's correlation coefficients were used to analyze the multicollinearity between the mRNAs and snoRNAs and CA19‐9.

## 3 Results

### 3.1 Intracellular distribution of exosomal RNAs in cultured PDAC cells

**Figure 1:**
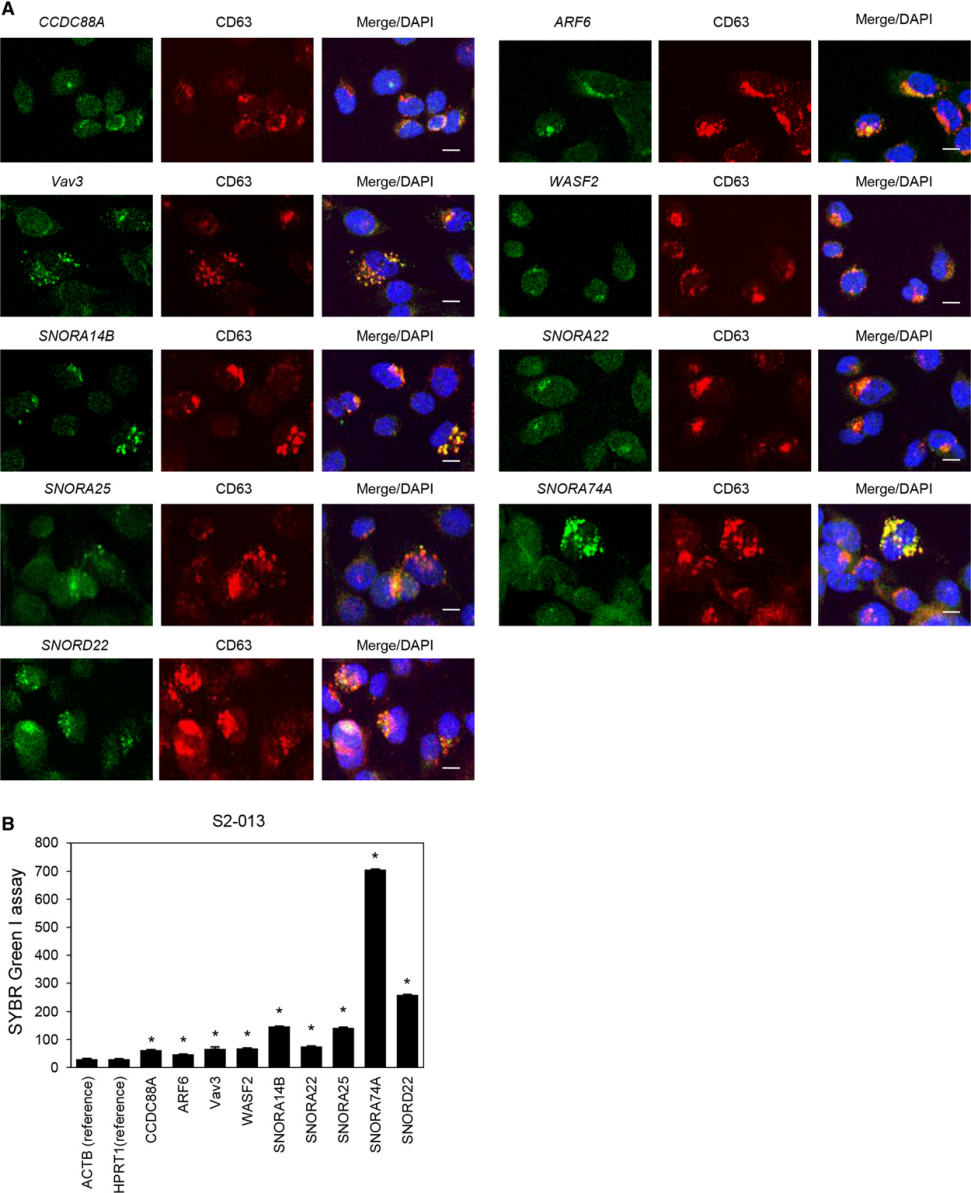
Intracellular distribution of exosomal RNAs in S2‐013 cells. (A) RNA fluorescence *in situ* hybridization images. S2‐013 cells were labeled with the probes from exosomal RNAs (green) and anti‐CD63 antibody (red). Blue, DAPI staining. Scale bars, 10 μm. (B) Exosomal RNAs were isolated from intracellular CD63‐positive exosomes of S2‐013 cells, and mRNAs for *CCDC88A*,*ARF6*,* Vav3*, and *WASF2* and snoRNAs for *SNORA14B*,*SNORA22*,*SNORA25*,*SNORA74A*, and *SNORD22* were measured by a SYBR Green I assay. Data are derived from three independent experiments. *Columns*, mean; *bars*, SD. *P < 0.0001 compared with reference RNAs for *ACT*B and *HPRT1* (Student's *t*‐test).

We used RNA fluorescence *in situ* hybridization to determine the subcellular localization of mRNAs for *CCDC88A*,* ARF6*,* Vav3*, and *WASF2* and snoRNAs for *SNORA14B*,* SNORA22*,* SNORA25*,* SNORA74A*, and *SNORD22* in moderately differentiated S2‐013 PDAC cells. All of these mRNAs and snoRNAs were concentrated in S2‐013 cells positive for the cytoplasmic exosome marker CD63 (Fig. 1A).

We validated the expression of these mRNAs and snoRNAs in the intracellular exosomes of S2‐013 cells. Intracellular CD63‐positive exosomes were isolated from S2‐013 cell lysates using an ExoCap Exosome composite kit. Intracellular exosomal RNAs were purified from the isolated CD63‐positive exosomes, and a SYBR Green I assay showed that all of these mRNAs and snoRNAs were significantly increased in CD63‐positive exosomes of S2‐013 cells compared to reference RNAs for *ACTB* and *HPRT1* (Fig. 1B).

### 3.2 Extracellular localization of exosomal RNAs from cultured PDAC cells

**Figure 2:**
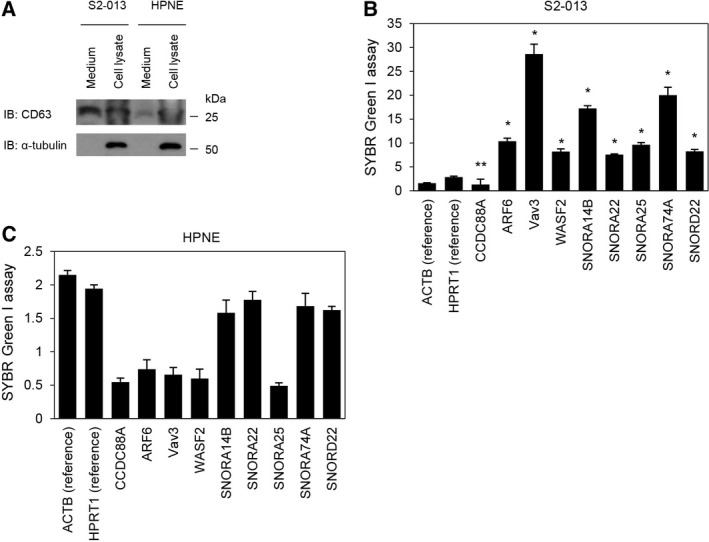
Extracellular localization of exosomal RNAs from S2‐013 and HPNE cells. (A) CD63 protein in total cell lysates and culture medium of S2‐013 and HPNE cells was examined by western blotting with an anti‐CD63 antibody probe. (B, C) Exosomal RNAs were isolated from conditioned medium harvested from S2‐013 (B) and HPNE (C) cells, and mRNAs for *CCDC88A*,*ARF6*,* Vav3*, and *WASF2* and snoRNAs for *SNORA14B*,*SNORA22*,*SNORA25*,*SNORA74A*, and *SNORD22* were measured by a SYBR Green I assay. Data are derived from three independent experiments. *Columns*, mean; *bars*, SD. *P < 0.0001 and **P = 0.098 compared with reference RNAs for *ACT*B and *HPRT1* (Student's *t*‐test).

We utilized a SYBR Green I assay to examine the presence of mRNAs for *CCDC88A*,* ARF6*,* Vav3*, and *WASF2* and snoRNAs for *SNORA14B*,* SNORA18*,* SNORA25*,* SNORA74A*, and *SNORD22* in CD63‐positive exosomes isolated from the culture medium of S2‐013 and HPNE cells. Western blotting showed the presence of CD63 in the total lysate and culture medium of both S2‐013 and HPNE cells (Fig. 2A). The expression level of CD63 was higher in the culture medium of S2‐013 cells compared with the medium of HPNE cells (Fig. 2A). The SYBR Green I assay revealed the presence of all of the mRNAs and snoRNAs except *CCDC88A* in CD63‐positive exosomes in S2‐013 cell culture medium (Fig. 2B). We found that levels of these RNAs in the culture medium of HPNE cells were lower than in the culture medium of S2‐013 cells (Fig. 2C). These results indicate that compared with HPNE cells, the expression of these RNAs was upregulated in exosomes secreted from S2‐013 cells.

### 3.3 Serum levels of exosomal RNAs

**Figure 3:**
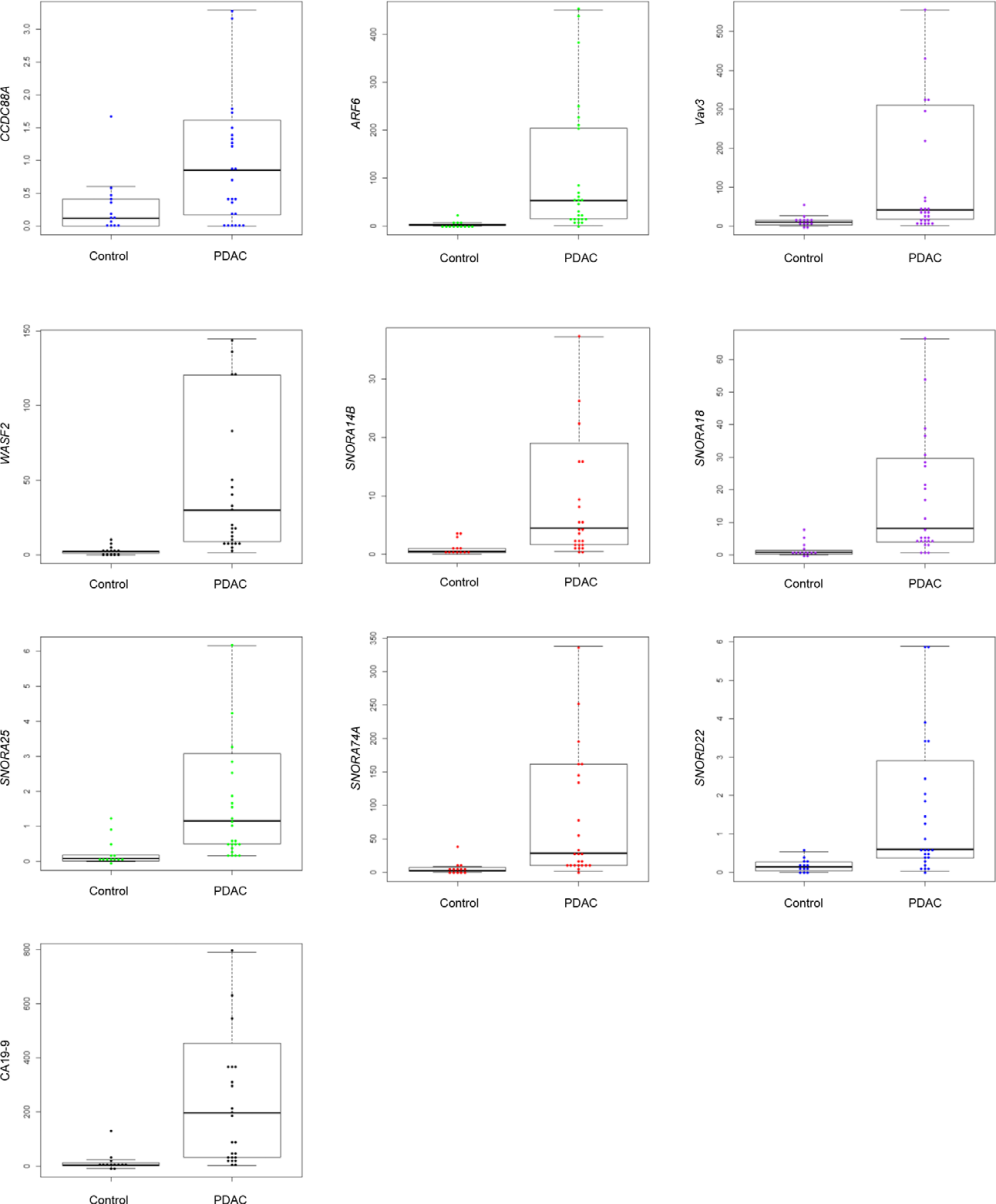
Serum levels of exosomal RNAs. Exosomal RNAs were isolated from patients with PDAC (*n* = 27) and disease‐free control patients (*n* = 13) and examined using a SYBR Green I assay. Pairwise comparisons of PDAC vs. control are shown. The horizontal line in the middle of each box indicates the median, whereas the top and bottom borders of the box mark the 75th and 25th percentiles, respectively. The upper whisker is the 75th percentile + (1.5 × interquartile range, IQR). The lower whisker is the 25th percentile − (1.5 × IQR).

The prospective clinical study included a total of 40 serological samples from patients with PDAC and control patients without pancreatic disease. The clinical characteristics of PDAC at the time of specimen procurement are summarized in Table 1. A SYBR Green I assay was used to compare the expression of four mRNAs (*CCDC88A*,* ARF6*,* Vav3*, and *WASF2*) and five snoRNAs (*SNORA14B*,* SNORA18*,* SNORA25*,* SNORA74A*, and *SNORD22*) in serum samples from patients with PDAC and disease‐free controls. The expression of all of the snoRNAs and mRNAs examined was significantly increased in pairwise comparisons between PDAC and control patients (*P* < 0.05; Fig. 3).

### 3.4 ROC curve analyses

**Figure 4:**
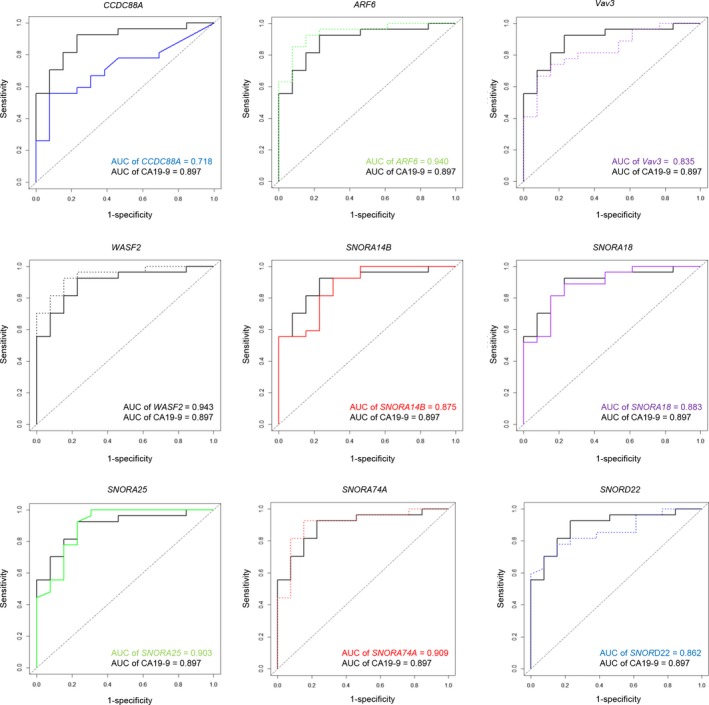
Diagnostic performance of the exosomal RNAs in distinguishing PDAC patients (*n* = 27) from control patients (*n* = 13), as determined using a SYBR Green I assay. ROC curves of levels of the exosomal RNAs and CA19‐9 in serum of PDAC patients and controls; *X*‐axis, 1 − specificity; *Y*‐axis, sensitivity.

Receiver operating characteristic curve analyses showed that two mRNAs (*WASF2* and *ARF6*) and two snoRNAs (*SNORA74A* and *SNORA25*) in serum provided excellent accuracy (AUC > 0.9) for distinguishing PDAC patients from controls (Fig. 4). The accuracy of each exosomal RNA in distinguishing PDAC from control patients is summarized by the AUC of ROC curves (Table 3). The AUC of CA19‐9 was 0.897 (95% CI, 0.797–0.997; Table 3).

**Table 3:** AUC values of ROC curves for four mRNAs, five snoRNAs, and CA19‐9

	AUC (95% CI)
*WASF2*	0.943 (0.875–1.000)
*ARF6*	0.940 (0.867–1.000)
*SNORA74A*	0.909 (0.807–1.000)
*SNORA25*	0.903 (0.795–1.000)
*SNORA22*	0.883 (0.774–0.993)
*SNORA14B*	0.875 (0.759–0.990)
*SNORD22*	0.862 (0.750–0.973)
*Vav3*	0.835 (0.707–0.962)
*CCDC88A*	0.718 (0.558–0.878)
CA19‐9	0.897 (0.797–0.997)

The relationships between the serum concentrations of these mRNAs (*WASF2* and *ARF6*) and snoRNAs (*SNORA74A* and *SNORA25*) and various clinicopathologic features were analyzed using the Wilcoxon rank‐sum test (Tables 4, 5, 6, 7). There were no significant associations between serum exosomal RNA concentrations and the clinical characteristics of age, gender, tumor size, or clinical stage in PDAC patients.

**Table 4:** Correlation between serum *WASF2* levels and clinicopathologic parameters in PDAC patients. IQR, interquartile range

Characteristic	Serum *WASF2* level		*P*‐value
	Median	IQR	
Age			
< 70 (*n* = 14)	17.2	8.5–138.7	0.905
≥ 70 (*n* = 13)	41.3	16.5–84.4	
Gender			
Male (*n* = 17)	29.8	7.1–84.4	0.264
Female (*n* = 10)	25.6	16.7–370.3	
Diagnosed with diabetes			
Yes (*n* = 13)	44.2	6.7–120.5	0.830
No (*n* = 14)	24.6	13.4–100.5	
Diagnosed with hypertension			
Yes (*n* = 11)	50.5	8.4–120.3	0.981
No (*n* = 16)	23.5	11.6–67.3	
Serum uric acid			
Upregulated (*n* = 2)	45.5	26.0–64.9	0.627
Normal range (*n* = 25)	29.8	9.8–120.5	
Serum triglyceride			
Upregulated (*n* = 8)	18.3	13.7–35.0	0.418
Normal range (*n* = 19)	41.3	8.0–140.7	
Stage^a^			
0, I, IIA (*n* = 8)	33.8	13.2–93.3	0.897
IIA, III (*n* = 19)	29.8	8.9–140.7	
Tumor diameter			
0–3 cm (*n* = 12)	40.1	8.1–130.3	0.999
> 3 cm (*n* = 15)	19.3	13.9–90.4	

^a^Classified according to the classification of International Union against Cancer. *P*‐values were calculated by the Wilcoxon rank‐sum test.

**Table 5:** Correlation between serum *ARF6* levels and clinicopathologic parameters in PDAC patients. IQR, interquartile range

Characteristic	Serum *ARF6* level		*P*‐value
	Median	IQR	
Age			
< 70 (*n* = 14)	52.5	17.9–240.4	0.402
≥ 70 (*n* = 13)	53.1	14.2–65.5	
Gender			
Male (*n* = 17)	47.0	15.3–86.5	0.414
Female (*n* = 10)	59.3	15.1–337.6	
Diagnosed with diabetes			
Yes (*n* = 13)	28.5	15.3–251.6	0.943
No (*n* = 14)	55.6	15.1–172.8	
Diagnosed with hypertension			
Yes (*n* = 11)	53.1	18.1–213.3	0.865
No (*n* = 16)	50.0	15.0–116.5	
Serum uric acid			
Upregulated (*n* = 2)	40.7	32.6–48.9	0.963
Normal range (*n* = 25)	53.1	14.2–206.8	
Serum triglyceride			
Upregulated (*n* = 8)	21.9	11.5–57.3	0.147
Normal range (*n* = 19)	53.1	18.1–238.4	
Stage^a^			
0, I, IIA (*n* = 8)	55.1	22.9–99.9	0.735
IIA, III (*n* = 19)	47.0	13.5–204.2	
Tumor diameter			
0–3 cm (*n* = 12)	55.1	22.8–207.5	0.323
> 3 cm (*n* = 15)	47.0	10.4–136.1	

^a^Classified according to the classification of International Union against Cancer.

**Table 6:** Correlation between serum *SNORA74A* levels and clinicopathologic parameters in PDAC patients. IQR, interquartile range

Characteristic	Serum *SNORA74A* level		*P*‐value
	Median	IQR	
Age			
< 70 (*n* = 14)	22.8	11.3–183.7	0.756
≥ 70 (*n* = 13)	56.4	10.4–161.7	
Gender			
Male (*n* = 17)	16.9	10.4–144.1	0.286
Female (*n* = 10)	56.8	25.8–302.8	
Diagnosed with diabetes			
Yes (*n* = 13)	56.4	10.8–162.2	0.905
No (*n* = 14)	27	11.0–141.1	
Diagnosed with hypertension			
Yes (*n* = 11)	144.1	12.7–179.5	0.544
No (*n* = 16)	26.3	10.7–93.2	
Serum uric acid			
Upregulated (*n* = 2)	130.0	69.2–190.8	0.999
Normal range (*n* = 25)	28.6	10.8–161.7	
Serum triglyceride			
Upregulated (*n* = 8)	31.5	24.7–62.2	0.979
Normal range (*n* = 19)	25.4	10.2–179.5	
Stage^a^			
0, I, IIA (*n* = 8)	95.2	22.9–170.8	0.549
IIA, III (*n* = 19)	25.4	10.6–139.4	
Tumor diameter			
0–3 cm (*n* = 12)	89.3	9.6–210.5	0.755
> 3 cm (*n* = 15)	28.6	12.4–107.0	

^a^Classified according to the classification of International Union against Cancer.

**Table 7:** Correlation between serum *SNORA25* levels and clinicopathologic parameters in PDAC patients. IQR, interquartile range

Characteristic	Serum *SNORA25* level		*P*‐value
	Median	IQR	
Age			
< 70 (*n* = 14)	1.4	0.5–5.3	0.610
≥ 70 (*n* = 13)	1.0	0.5–2.5	
Gender			
Male (*n* = 17)	1.2	0.5–5.3	0.900
Female (*n* = 10)	0.9	0.5–5.3	
Diagnosed with diabetes			
Yes (*n* = 13)	1.6	0.5–3.3	0.357
No (*n* = 14)	0.8	0.4–2.4	
Diagnosed with hypertension			
Yes (*n* = 11)	1.7	0.5–3.1	0.300
No (*n* = 16)	0.8	0.3–2.5	
Serum uric acid			
Upregulated (*n* = 2)	1.9	1.2–2.6	0.746
Normal range (*n* = 25)	1.2	0.5–2.8	
Serum triglyceride			
Upregulated (*n* = 8)	0.6	0.5–1.1	0.232
Normal range (*n* = 19)	1.7	0.4–5.2	
Stage^a^			
0, I, IIA (*n* = 8)	1.1	0.5–2.6	0.958
IIA, III (*n* = 19)	1.2	0.4–5.2	
Tumor diameter			
0–3 cm (*n* = 12)	1.7	0.5–3.0	0.922
> 3 cm (*n* = 15)	1.0	0.5–2.9	

^a^Classified according to the classification of International Union against Cancer. *P*‐values were calculated by the Wilcoxon rank‐sum test.

### 3.5 Association between RNA levels and risk of PDAC

The significance of differences in the serum levels of two mRNAs (*WASF2* and *ARF6*) and two snoRNAs (*SNORA74A* and *SNORA25*) in terms of PDAC diagnosis was evaluated using logistic regression to obtain crude odds ratios (ORs; Table 8). Crude ORs adjusted for CA19‐9 were then adjusted to exclude possible effects of age and gender. The results suggested that the serum level of *WASF2* mRNA was the most highly correlated with the risk of PDAC.

**Table 8:** Univariate and multivariate logistic regression analyses. CA19‐9, cancer antigen 19‐9; Model 1: odds ratio adjusted for CA19‐9; Model 2: odds ratio adjusted for CA19‐9, age, and gender

Indicators	Crude OR	*P*‐value	Adjusted OR of model 1	*P*‐value	Adjusted OR of model 2	*P*‐value
*WASF2*	1.54 (95% CI: 1.11–2.14)	0.009	*WASF2*1.51 (95% CI: 1.07–2.12)	0.019	*WASF2*2.45 (95% CI: 0.77–7.74)	0.187
			CA19‐91.02 (95% CI: 0.99–1.05)	0.183	CA19‐91.09 (95% CI: 0.96–1.24)	0.196
*ARF6*	1.12 (95% CI: 1.00–1.43)	0.013	*ARF6*1.30 (95% CI: 1.02–1.64)	0.033	*ARF6*1.72 (95% CI: 0.64–4.63)	0.284
			CA19‐91.03 (95% CI: 0.99–1.06)	0.104	CA19‐91.15 (95% CI: 0.86–1.54)	0.359
*SNORA74A*	1.22 (95% CI: 1.04–1.25)	0.050	*SNORA74A*1.10 (95% CI: 0.98–1.24)	0.099	*SNORA74A*1.13 (95% CI: 0.96–1.33)	0.136
			CA19‐91.02 (95% CI: 0.99–1.04)	0.165	CA19‐91.03 (95% CI: 0.99–1.08)	0.184
*SNORA25*	12.7 (95% CI: 1.3‐124)	0.029	*SNORA25*8.87 (95% CI: 0.95–82.1)	0.055	*SNORA25*6.23 (95% CI: 0.59–65.6)	0.284
			CA19‐91.02 (95% CI: 0.99–1.04)	0.159	CA19‐91.02 (95% CI: 0.99–1.05)	0.161
CA19‐9	1.02 (95% CI: 1.00–1.05)	0.073	–	–	1.03 (95% CI: 1.00–1.06)	0.041

### 3.6 Detection of early‐stage PDAC using exosomal RNAs and CA19‐9

**Figure 5:**
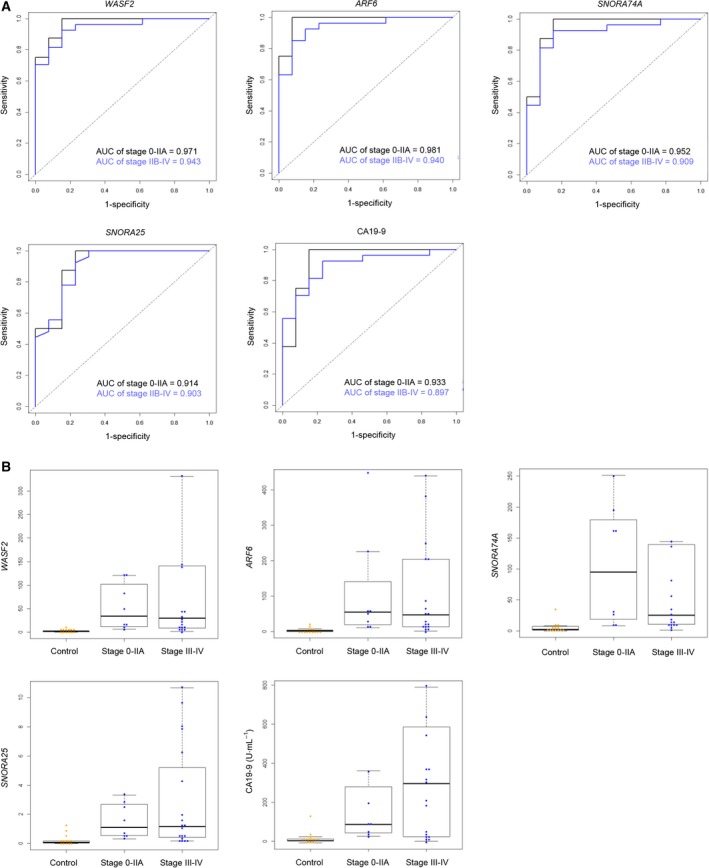
Diagnostic performance of the exosomal RNAs in distinguishing stage 0/I/IIA and stage IIB/III/IV PDAC, determined using a SYBR Green I assay. (A) ROC curves of levels of two mRNAs (*WASF2* and *ARF6*), two snoRNAs (*SNORA74A* and *SNORA25*), and CA19‐9 in serum of stage 0/I/IIA PDAC patients (*n* = 8), stage IIB/III/IV PDAC patients (*n* = 19), and controls (*n* = 13); *X*‐axis, 1 − specificity; *Y*‐axis, sensitivity. (B) Levels of two mRNAs (*WASF2* and *ARF6*), two snoRNAs (*SNORA74A* and *SNORA25*), and CA19‐9 in serum of stage 0/I/IIA PDAC patients (*n* = 8), stage IIB/III/IV PDAC patients (*n* = 19), and controls (*n* = 13). The horizontal line in the middle of each box indicates the median, whereas the top and bottom borders of the box mark the 75th and 25th percentiles, respectively. The upper whisker is the 75th percentile + (1.5 × interquartile range, IQR). The lower whisker is the 25th percentile − (1.5 × IQR).

The AUC values of ROC curves for five snoRNAs, four mRNAs, and CA19‐9 are summarized in Table 9. Two mRNAs (*WASF2* and *ARF6*) and two snoRNAs (*SNORA74A* and *SNORA25*) for distinguishing patients with stage 0/I/IIA and stage IIB/III/IV PDAC from disease‐free controls had values > 0.9 (Table 9). The AUC values of ROC curves for CA19‐9 for distinguishing patients with stage 0/I/IIA and stage IIB/III/IV PDAC from controls were 0.933 and 0.897, respectively (Table 9, Fig. 5A). The levels of *WASF2*,* ARF6*,* SNORA74A*,* SNORA25*, and CA19‐9 in serum samples from patients in the early stages of PDAC (stages 0, I, and IIA) were significantly higher than the level in controls (Fig. 5B). Interestingly, the AUC values of ROC curves for *SNORA22* and *SNORD22* in patients with stage 0/I/IIA PDAC were higher than those in patients with stage IIB/III/IV PDAC.

**Table 9:** AUCs of ROC curves for four mRNAs, five snoRNAs, and CA19‐9 in distinguishing stage 0/I/IIA and stage IIB/III/IV PDAC

	AUC score (95% CI) in stage 0/I/IIA	AUC score (95% CI) in stage IIB/III/IV
*WASF2*	0.971 (0.914–1.000)	0.944 (0.875–1.000)
*ARF6*	0.981 (0.936–1.000)	0.940 (0.867–1.000)
*SNORA74A*	0.952 (0.866–1.000)	0.909 (0.807–1.000)
*SNORA25*	0.914 (0.792–1.000)	0.903 (0.795–1.000)
*SNORA22*	0.914 (0.792–1.000)	0.883 (0.774–0.993)
*SNORA14B*	0.856 (0.691–1.000)	0.875 (0.759–0.990)
*SNORD22*	0.966 (0.904–1.000)	0.862 (0.750–0.973)
*Vav3*	0.885 (0.721–1.000)	0.835 (0.707–0.962)
*CCDC88A*	0.726 (0.489–0.963)	0.718 (0.558–0.878)
CA19‐9	0.933 (0.825–1.000)	0.897 (0.797–0.997)

### 3.7 Combination of exosomal RNAs with CA19‐9

**Figure 6:**
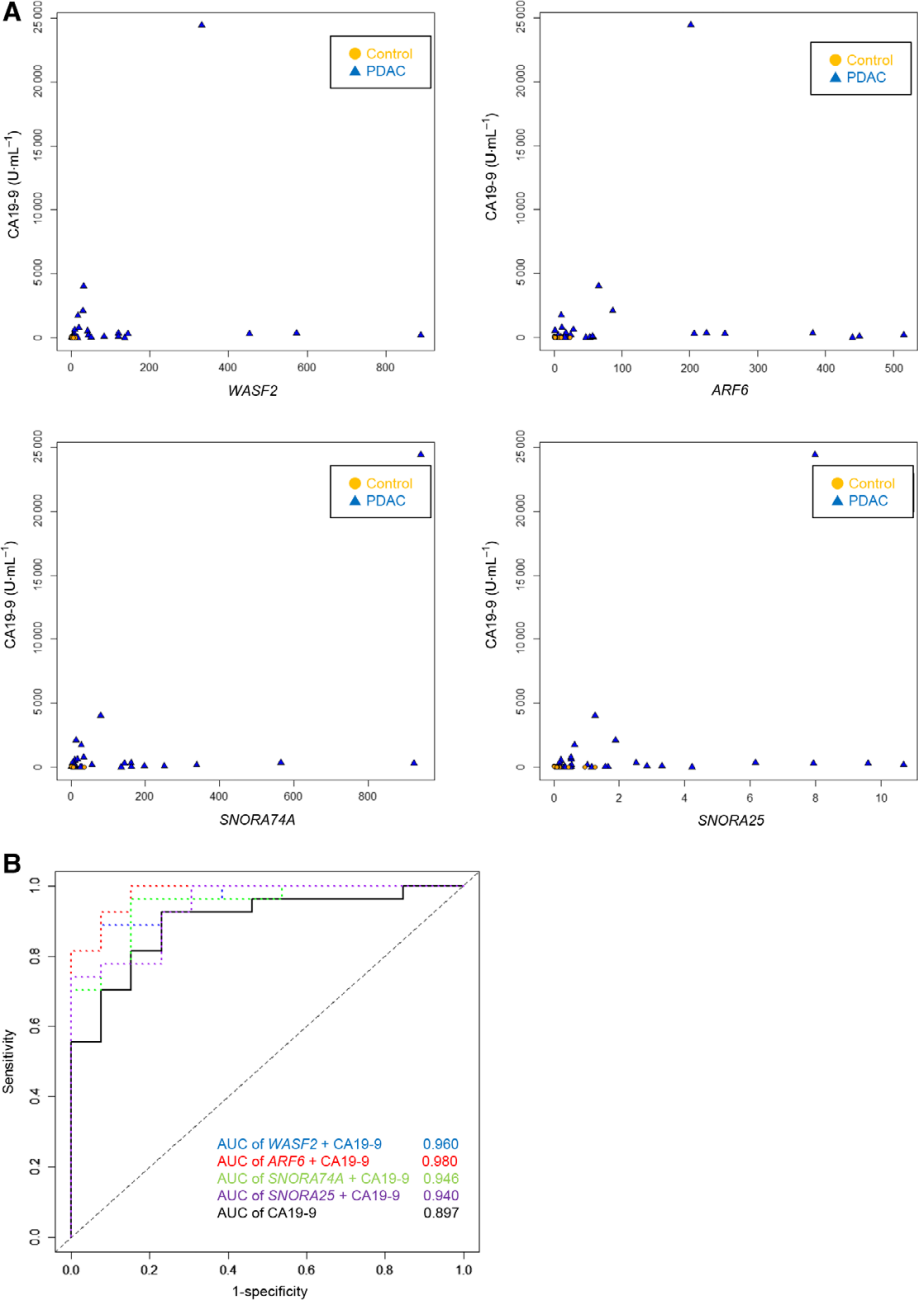
Performance of the combination of exosomal RNAs and CA19‐9 for distinguishing PDAC patients from controls, as determined using a SYBR Green I assay. (A) Relationship between serum levels of two mRNAs (*WASF2* and *ARF6*) and two snoRNAs (*SNORA74A* and *SNORA25*) and CA19‐9 in PDAC patients (*n* = 27; *X*‐axis, CA19‐9 concentration; *Y*‐axis, exosomal RNA concentration). (B) ROC curves of serum levels of two mRNAs (*WASF2* and *ARF6*) and two snoRNAs (*SNORA74A* and *SNORA25*) in combination with CA19‐9 for distinguishing PDAC patients (*n* = 27) from controls (*n* = 13); *X*‐axis, 1 − specificity; *Y*‐axis, sensitivity.

Expression data for two mRNAs (*WASF2* and *ARF6*), two snoRNAs (*SNORA74A* and *SNORA25*), and CA19‐9 were combined in an attempt to improve the sensitivity and specificity of the markers for detecting PDAC. To account for multicollinearity in the logit models of these exosomal RNAs and CA19‐9, Pearson's correlation coefficient was employed. There were no significant correlations between the serum levels of the RNAs and CA19‐9; the Pearson's correlation coefficient (*R*) was 0.221 (95% CI, 0.100–0.500) between *WASF2* and CA19‐9; 0.136 (95% CI, 0.183–0.429) between *ARF6* and CA19‐9; 0.600 (95% CI, 0.354–0.768) between *SNORA74A* and CA19‐9; and 0.358 (95% CI, 0.053–0.603) between *SNORA25* and CA19‐9 (Fig. 6A).

Receiver operating characteristic curve analysis of the combinations returned AUC values of 0.960 (95% CI, 0.910–1.000) for the combination of *WASF2* with CA19‐9; 0.980 (95% CI, 0.946–1.000) for the combination of *ARF6* with CA19‐9; 0.946 (95% CI, 0.879–1.000) for the combination of *SNORA74A* with CA19‐9; and 0.940 (95% CI, 0.871–1.000) for the combination of *SNORA25* with CA19‐9 (Fig. 6B). These AUC values were higher than those for the RNAs or CA19‐9 alone as shown in Table 3.

## 4 Discussion

To decrease the mortality caused by PDAC, efficient screening methods capable of detecting the disease in the early stages are needed (Marengo and Robotti, 2014). This study describes a novel panel of serum exosomal RNAs, including four mRNAs and five snoRNAs, for use in diagnosing PDAC. We demonstrated that the measurement of these serum exosomal RNAs enables detection of the early stages of PDAC. The AUC value of the ROC curve for levels of the current standard marker, CA19‐9, in serum of patients in the early stages of PDAC (stages 0, I, and IIA) was 0.93 (Table 3), but the use of this marker is limited to monitoring the response to therapy rather than diagnosing the early stages of PDAC (DiMagno *et al*., 1999).

We recently reported that an RNA‐binding protein, insulin‐like growth factor‐2 mRNA‐binding protein 3 (IGF2BP3), and IGF2BP3‐bound RNAs localize in cytoplasmic RNA granules that accumulate in the membrane protrusions of PDAC cells (Taniuchi *et al*., 2014a,b). Local translation of IGF2BP3‐bound mRNAs induces formation of the protrusions, thereby promoting the motility, invasion, and metastasis of cancer cells (Taniuchi *et al*., 2014a,b). The mRNAs for *CCDC88A*,* ARF6*,* Vav3*, and *WASF2* bind to IGF2BP3 (Taniuchi *et al*., 2014a), and CCDC88A, ARF6, and Vav3 proteins accumulated in membrane protrusions promote the motility and invasion of PDAC cells (Taniuchi *et al*., 2014a; Tanouchi *et al*., 2016; Tsuboi *et al*., 2016). snoRNAs for *SNORA14B*,* SNORA18*,* SNORA25*,* SNORA74A*, and *SNORD22* also bind IGF2BP3 in PDAC cells (Taniuchi *et al*., 2014a); however, the mechanisms by which IGF2BP3‐bound mRNAs and snoRNAs are transferred from cytoplasmic RNA granules to intracellular exosomes are unknown. The mechanism underlying the packaging process and its importance is thus an important subject for future studies.

Small nucleolar RNAs are precursors for functional small RNAs, and snoRNA‐derived small RNAs can function like microRNAs (Ender *et al*., 2008). The snoRNA *ACA45* is processed into 20–25 small nucleotide RNAs, and *ACA45*‐processing RNAs bound to Ago proteins contribute to posttranscriptional gene silencing of target mRNAs (Ender *et al*., 2008). The U/A‐rich *SNORD50A* inhibits 3′‐processing of its target mRNAs by blocking interactions with the Fip1‐poly(A) site, resulting in changes in alternative polyadenylation profiles and/or posttranslational regulation of subsets of target mRNAs (Huang *et al*., 2017). As the functional roles of snoRNAs in PDAC remain unclear, future studies will explore whether the snoRNAs analyzed in this study are functionally linked to posttranscriptional regulation and the motility and invasiveness of PDAC cells.

A SYBR Green I assay showed that mRNAs for *CCDC88A*,* ARF6*,* Vav3*, and *WASF2* and snoRNAs for *SNORA14B*,* SNORA22*,* SNORA25*,* SNORA74A*, and *SNORD22* were present in intracellular exosomes of S2‐013 cells. All these mRNAs and snoRNAs except for *CCDC88A* mRNA were secreted and present in extracellular exosomes of cell culture medium harvested from S2‐013 cells. The SNARE protein YKT6, which is targeted and regulated by miR‐134 and miR‐135b, regulates exosome release in lung cancer cells (Ruiz‐Martinez *et al*., 2016). Suppression of YKT6 reduces exosome release from lung cancer cells (Ruiz‐Martinez *et al*., 2016). Since exosomes are highly heterogeneous (Kowal *et al*., 2016) and the expression of *CCDC88A* mRNA was significantly increased in serological samples from PDAC patients, it is possible that a key molecule may regulate *CCDC88A* mRNA‐containing exosome release from S2‐013 cells. The serum concentration and composition of exosomes are altered by different pathophysiological conditions (Patel *et al*., 2016). Hypoxic conditions in the tumor microenvironment promote tumor metastasis by altering exosome release to regulate cell–cell communication (Ackerman and Simon, 2014; Lu and Kang, 2010). Although the process of how particularly selected exosomes are secreted from PDAC cells is largely unknown, it is likely that pathophysiological conditions affect exosome release from PDAC cells. It is therefore predicted that the serum level of *CCDC88A* mRNA would not be upregulated in PDAC patients whose PDAC tumors contain a high population of PDAC cells, such as S2‐013, that have inhibited release of *CCDC88A* mRNA‐containing exosomes. In contrast, the serum level of *CCDC88A* mRNA would be upregulated in PDAC patients with a low population of PDAC cells that have inhibited release of *CCDC88A* mRNA‐containing exosomes. The PDAC‐specific mechanisms by which key molecules regulate exosome release and the particular exosomal RNAs that are secreted are important subjects for the identification of diagnostic markers associated with PDAC exosomes.

The number of exosomes is elevated in the systemic circulation of patients with PDAC (Melo *et al*., 2015). Exosomes from cancer cells secrete mRNAs, microRNAs, snoRNAs, and lncRNAs into body fluids such as the blood, urine, milk, and saliva (Laurent *et al*., 2015). The isolation of exosomes from cancer cells could lead to the identification of specific diagnostic markers capable of distinguishing exosomes from cancerous and noncancerous cells (Melo *et al*., 2015). Levels of the microRNA *17‐5p* are elevated in serum exosomes of PDAC patients, and increased *17‐5p* microRNA expression is positively correlated with metastasis and staging (Que *et al*., 2013). h*TERT* mRNA, the transcript of the enzyme telomerase, is shuttled from PDAC cells via exosomes into telomerase‐negative fibroblasts; however, whether h*TERT* mRNA is present in serum PDAC‐derived exosomes remains unclear (Gutkin *et al*., 2016). Serum concentrations of the snoRNA *SNORD91B* are decreased in PDAC patients (Liu *et al*., 2014), and to date, there is no evidence indicating upregulation of snoRNA expression in PDAC development. To the best of our knowledge, this study is the first report of elevated snoRNA levels in the circulation of PDAC patients.

It is important to identify PDAC‐specific circulating mRNAs and snoRNAs that could be highly sensitive and specific serum markers of this disease. Although the sample size of this study was limited, the present study demonstrated that ORs adjusted for CA19‐9 showed that the serum level of *WASF2* mRNA was the most highly correlated with the risk of PDAC (Table 8), and the lower limits of the 95% CI of the AUCs were relatively high, especially those of *WASF2* and *ARF6* (0.875 and 0.867, respectively; Table 3), and distribution bias was not detected between the early stages of PDAC (stages 0, I, and IIA) and the late stages of PDAC (stages IIB and III; Fig. 5B). Thus, the present study provides the statistically meaningful finding that analyzing serum levels of *SNORA74A*,* SNORA25*,* WASF2*, and *ARF6* would be a useful novel approach for distinguishing patients with PDAC from patients without the disease. More extensive studies aimed at clarifying the potential superiority of *SNORA74A*,* SNORA25*,* WASF2*, and *ARF6* to CA19‐9 in distinguishing patients with stage 0/I/IIA PDAC from controls are needed. Additionally, the combination of measuring the levels of one of these RNAs along with that of CA19‐9 may be superior to the use of CA19‐9 alone in establishing a diagnosis of PDAC. Toward this end, we have started two prospective clinical validation studies (UMIN #000021938 and UMIN #000031970) at the Kochi Medical School Hospital, Kochi Health Sciences Center, Hata Prefectural Hospital, and Kanagawa Cancer Center to assess the utility of *SNORA74A*,* SNORA25*,* WASF2*, and *ARF6* as potential diagnostic markers for the early detection of PDAC in comparison with CA19‐9.

## 5 Conclusions

The present study revealed that *WASF2*,* ARF6*,* SNORA74A*, and *SNORA25* may be novel, noninvasive diagnostic biomarkers for the early detection of PDAC. In particular, monitoring serum levels of *WASF2* mRNA may be useful, as it was the most highly correlated with PDAC risk. These findings should be validated in large‐scale prospective clinical studies to use them as diagnostic markers for the detection of PDAC.

## Conflict of interest

The authors declare no conflict of interest.

## Author contributions

KT designed and performed the experiments, analyzed the data, wrote the manuscript with contributions from all authors, and obtained financial support; TK, MT and TO performed experiments; MS performed statistical analyses; and TS analyzed the data and supervised the research.
